# Supplementary material for: A novel hybrid model for emotion detection in text through sequential and transformer-based approaches: LSTM enhanced RoBERTa (LER)
Source: Sci Rep. 2026 Jan 19;16:2224. doi: 10.1038/s41598-025-31984-1 (PMC12816582; doi:10.1038/s41598-025-31984-1)
Supplement: Supplementary file 1 — Supplementary Material 1 [file 41598_2025_31984_MOESM1_ESM.docx]

A Novel Hybrid Model for Emotion Detection in Text through Sequential and Transformer-Based Approaches: LSTM Enhanced RoBERTa (LER)

Bilal Khan^1^[0000-0002-6816-3776], Muhammad Usman^2^[0000-0001-8363-0179] and Muhammad Binsawad^3^[0000-0003-0915-7058]

^1^ Department of Computer Software Engineering, University of Engineering and Technology, Mardan, 23200, Pakistan, bilalsoft63@gmail.com

^2^ Department of Computer Science, University of Engineering and Technology, Mardan, 23200, Pakistan, usman@uetmardan.edu.pk

^3^ Department of Information Systems, Faculty of Computing and Information Technology, King Abdulaziz University, Jeddah, 21589, Saudi Arabia, mbinsawad@kau.edu.sa

**Abstract.** Text emotion detection is an essential task in Natural Language Processing (NLP), with applications in customer support automation, diagnosing mental health, and social media analysis. Yet, precise emotion detection is a difficult problem as human emotional states are subtle, ambiguous, and contextual. Current models typically fail to fully grasp these subtleties. To overcome these limitations, the current study proposes a new hybrid architecture, LSTM Enhanced RoBERTa (LER), that combines the sequential learning ability of Long Short-Term Memory (LSTM) networks with the deep contextual knowledge provided by the RoBERTa transformer model. The LER model proposed here is tested on the popular ISEAR emotion dataset and registers an impressive accuracy of 88%, which surpasses many robust baseline models. The model's performance is evaluated on standard metrics, such as precision, recall, and F1-score. The results show that the hybrid approach efficiently detects intricate emotional cues, thus improving the state of emotion detection for real-world, context-sensitive applications.

**Keywords:** Emotion Detection, NLP, Sequence Learning, Transformer Models, Sentiment Analysis, Text Classification.

1. Introduction

In today’s fast-paced world, social media platforms are ubiquitous, enabling effortless communication via text, video, and voice. Emotions are central to these interactions, helping users express themselves and build deeper connections (Wang et al. 2012; Chatterjee et al. 2019). While emotions can be effectively conveyed through facial expressions, body language, and tone, interpreting them in written text remains challenging due to the ambiguity of language (Sailunaz et al. 2018; Rodríguez et al. 2020).

Emotions are foundational to both direct and indirect communication, including psychosocial systems used in studying criminal behavior (Dennison 2024). Human expression—through words, gestures, and vocal cues—reflects emotional states (Chen et al. 2018). Though progress has been made in recognizing emotions from speech and facial expressions, text-based emotion recognition is still underdeveloped (Ezzameli and Mahersia 2023; Waelen 2024). Detecting emotions such as joy, anger, sadness, and fear is important in applications like analytics and language modeling (Asghar et al. 2021), yet the concept of emotions remains fragmented across cognitive science (Hasan et al. 2019). Psychology emphasizes understanding emotions due to their impact on perception, decisions, relationships, and mental health (Hossain and Muhammad 2019). Emotional intelligence is also vital in healthcare, law, marketing, and education (Velagaleti et al. 2024).

Advancing text-based emotion detection enhances human understanding. NLP techniques help identify emotional patterns, while ML and DL models determine sentiment, supporting domains such as mental health, customer service, opinion mining, and personalization (Al Maruf et al. 2024). However, detecting emotion in informal, user-generated text—often short, ungrammatical, and filled with typos, slang, or symbols—poses major challenges, requiring advanced preprocessing and robust feature extraction (Chutia and Baruah 2024).

To address these limitations, this research proposes a novel model: LSTM-Enhanced RoBERTa (LER), which integrates Long Short-Term Memory (LSTM) with the transformer-based RoBERTa model. The model is evaluated against state-of-the-art ML and DL techniques—Random Forest (RF), Support Vector Machine (SVM), Logistic Regression (LR), Naïve Bayes (NB), XGBoost (XGB), Convolutional Neural Network (CNN), Gated Recurrent Unit (GRU), Bi-GRU, BERT, LSTM, and RoBERTa—using precision, recall, F1-score, and accuracy.

LER’s primary contribution lies in effectively capturing sequential dependencies and contextual nuances by combining LSTM’s temporal modeling with RoBERTa’s deep language understanding. Through rigorous testing, it demonstrates superior accuracy and performance over traditional methods. This advancement is especially valuable for applications in mental health monitoring, sentiment tracking, and social media analysis, and lays the groundwork for future specialized models with enhanced emotion recognition and contextual comprehension.

The novelty of the proposed LSTM-Enhanced RoBERTa (LER) model lies in its integration of contextual embeddings with sequential refinement through a BiLSTM layer, combined with optimized hyperparameters and regularization strategies to balance accuracy and efficiency. Unlike earlier hybrid approaches, LER explicitly models temporal dependencies on top of RoBERTa’s deep contextual representations and incorporates uncertainty analysis to assess robustness. This design yields significant improvements across all performance metrics (accuracy 88%, precision 86.03%, recall 85.21%, F1 86.9), demonstrating the model’s superiority for emotion detection compared to both traditional ML and recent DL baselines.

The remainder of this study is organized as follows: Section 2 delves into the background of human emotion detection, offering important context and exploring various methodologies in this exciting field. Section 3 introduces our proposed scheme, detailing the dataset we utilized, the preprocessing techniques we implemented, and the methods we employed for feature extraction. This section also walks through our model training process and performance evaluation, while comparing our approach to established benchmark machine learning and deep learning models. Section 4 presents the results of our experiments, highlighting their significance and implications. Finally, in Section 5, we wrap up the study with conclusions and offer insights into future research directions in human emotion detection.

1. Literature Review

Various theories and models were developed through extensive research in affective science in emotion detection and classification. The emotion classification models can be categorized into two categories, which are discrete and dimensional (Alslaity and Orji 2024). In discrete models, emotions are identified within specific classes. For example, Plutchik's model (Plutchik 1980) mentions eight primary emotions, such as sadness, fear, surprise, anger, disgust, trust, anticipation, and joy, which it depicts on the "Wheel of Emotions." Yet another is Ekman's model (Ekman 1992), which states that six basic emotions, including fear, disgust, anger, sadness, happiness, and surprise, are broadly believed to be recognizable across cultures (Alslaity and Orji 2024). In dimensional models, emotions are categorized along dimensions rather than fixed categories. A dimensional theory of emotion organizes emotions within a dimensional affective space, which has dimensions such as arousal, valence, and intensity (Lee et al. 2012). Most early studies make use of three dimensions; however, many recent studies make use of two-dimensional models (Fontaine et al. 2007). Currently, there is no compromise on the number of dimensions required to capture emotion adequately. Some of the dimensional models include the Circumplex model (Russell 1980); the Positive Affect Negative Affect Scale, PANAS (Watson et al. 1988); and the Pleasure, Arousal, and Dominance, PAD model (Mehrabian 1996).

As emotion detection systems can be categorized as discrete or multi-dimensional characteristics (PS and Mahalakshmi 2017), using the discrete emotion models can detect fine-grained categories, including anger, fear, sadness, joy, surprise, disgust, depression, and love. Two common models are Ekman's six-emotion model (Ekman 1999) and Plutchik's eight-emotion model (Plutchik 1980). Alternatively, multi-dimensional models of emotion evaluation are based on dimensions like valence, arousal, and power that treat emotions as interconnected. Some of these models are used for the analysis of polarity, activation/deactivation, and emotion intensity, and some of the widely used multi-dimensional models.

Many studies exist with the comparison of ML models for emotion detection and sentiment analysis. This involves various examples, which compare (Narendra et al. 2016) a user's review of the movie concerning an ML model as compared to Nabil et al. on a comparison among algorithms of ML algorithms from an Amazon review. Other research works have compared different ML models for sentiment analysis in domains such as film reviews in the Gujarati language (Shah and Swaminarayan 2022), social media texts (Hammad and Al-Awadi 2016; Singh et al. 2020), and news articles (Vaseeharan and Aponso 2020). This study does not give an overview of the latest ML techniques applied to sentiment analysis and emotion detection. Some researchers have worked on systematic reviews to investigate some of the aspects of ML in emotion detection and sentiment analysis. For instance, Machado et al. published a literature review on the application of ML techniques to text-based sentiment analysis for reviews, comments, and evaluations (Machado and Ribeiro 2019). This research overviewed the trend in publication but did not detail some of the ML-related aspects.

Seal et al. (Al Maruf et al. 2024) have utilized a keyword-based method primarily concentrating on phrasal verbs to accomplish emotion recognition. After preprocessing the data using ISEAR (Alnuaim et al. 2022), they used the keyword-based technique. They created their database after finding several phrasal verbs that ought to have been connected to emotion categories but weren't. Using their database, they classified phrasal verbs and keywords that were associated with different emotions. They were able to address the researcher's existing problems, such as an inadequate list of emotion keywords and a disregard for word semantics in meaning, even if they did obtain a considerably greater accuracy of 65%. Based on the convolutional neural network (CNN), Dongliang Xu et al. (Xu et al. 2020) created the CNN-Text_Word2vec microblog emotion categorization model. In addition to successfully extracting the important features, the approach produced a good classification effect. The experiment's findings demonstrated that the scheme's emotional classification accuracy outperforms SVM, RNN, and LSTM by 7%, 6.9%, and 2.91%, respectively. However, it was limited by the features' incorrect ranking.

Based on the sentiments expressed in social media conversations, politicians may be able to comprehend the public's worries about security-related issues. Marketers can create sales agendas to promote their products and services (Chung and Zeng 2020). Twitter is a significant social media platform that has been rebranded as "X." The authors focused on Twitter data to analyze historical and current feeds to extract emotions, as stated hashtags on Twitter postings can carry different semantic payloads. Researchers downloaded Arabic texts from the social media site Twitter, and then human annotators gave each text the proper emotions (Cassab and Kurdy 2020). By identifying subtle emotions in online health groups, one might obtain insight into patients' emotional circumstances and so understand their emotional states.

Outside the realm of emotional classification, neural networks, and more specifically recurrent versions, have shown remarkable usefulness in other contexts. For example, dynamic recurrent functional link neural networks have been used for nonlinear system identification with Lyapunov stability analysis. Diagonal recurrent neural networks and self-recurrent wavelet neural networks have also been effectively deployed for adaptive control as well as predictive modeling of nonlinear dynamical systems. These uses underscore the general utility of neural models in both cognitive and control systems environments. Neural networks have performed remarkably well in challenging control and system identification problems, mostly because they can handle temporal dependencies and represent nonlinear dynamics well. Notable applications include:

1. *Dynamic Recurrent Functional Link Neural Networks (DRFLNN)* used for nonlinear system identification, where Lyapunov stability analysis ensures robust convergence and system stability.

2. *Diagonal Recurrent Neural Networks (DRNN)* are employed for adaptive control of nonlinear dynamical systems, leveraging Lyapunov-based stability criteria to maintain reliable and stable control performance.

3. *Self-Recurrent Wavelet Neural Networks*, which integrate temporal memory with multiresolution wavelet functions, are applied for both identification and adaptive predictive control in highly nonlinear and time-varying environments.

These developments highlight the flexibility of neural networks in areas like robotics, industrial process control, and autonomous systems. By adding this wider context, we hope to highlight the underlying strength of neural architectures such as LSTM and their applicability in both cognitive computing (e.g., emotion recognition) and control systems.

Recent developments in object recognition-based real-time emotion detection techniques, especially for humanoid robots, have brought about advanced methods that can help improve the emotion detection functionality outlined in the proposed LSTM-Enhanced RoBERTa (LER) model for text-based emotion detection. Although the LER model, as described in the research paper, is highly accurate at 88% on the ISEAR dataset by tapping into RoBERTa's contextual embeddings and LSTM's sequential processing of text data (Section: Results and Discussion), it considers only text and not multimodal inputs such as facial expressions or body movements, which are imperative for real-time human-robot interaction (HRI). A good example of such developments is the emotion analysis algorithm deployed on the NAO humanoid robot that employs a four-step process based on the Viola-Jones algorithm for facial detection, geometric-based facial distance measurement, and the Facial Action Coding System (FACS) to analyze emotional attributes from facial expressions with good reliability in emotion recognition with tested accuracy, computational load, and speed. This real-time HRI algorithm differs from the LER model's text-based methodology, pointing to the possibility of expanding on LER with the inclusion of visual processing of data, i.e., CNNs for face landmark localization, to recognize non-verbal emotional indicators. Integrating such object recognition-based methods, as proposed in the Conclusion and Future Directions, may make LER more applicable to multimodal HRI applications, such as mental health diagnosis or social robotics, by integrating textual analysis with real-time facial and body movement recognition for more complete emotion detection.

1. Proposed Scheme

This study aims to propose a hybrid model, LSTM-Enhanced RoBERTa (LER), for emotion detection in textual data, which can facilitate us in various fields of study, including mental health monitoring, customer feedback analysis, social media sentiment analysis, personalized marketing, educational technology for student engagement assessment, and human-computer interaction for adaptive systems. The dataset used in this study has been taken from an online repository, which is discussed in the subsequent section. The overall methodology of this study is presented in Figure 1, followed by preprocessing, model training, and testing for model performance evaluation. Each phase is further discussed in the subsequent section for better understanding.


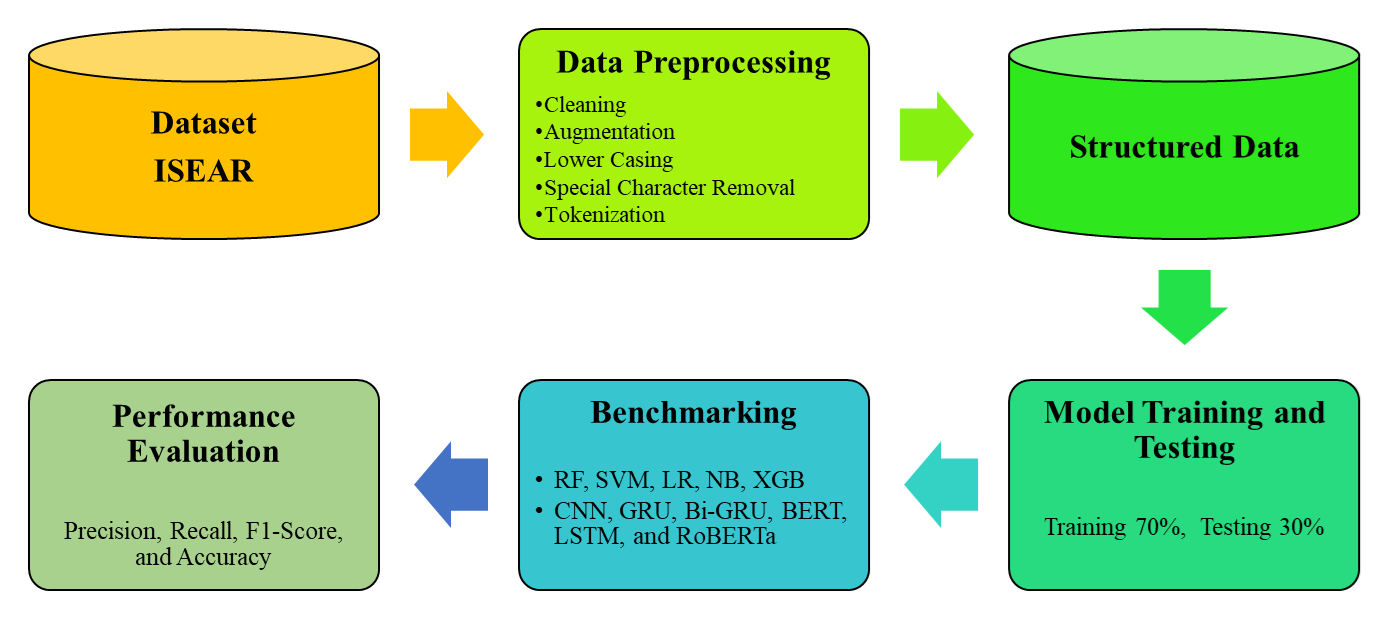


**Fig. 1.** Overview of the Proposed Research Workflow for Emotion Detection.

- 1. Dataset Description and Preprocessing

The dataset used in this study has been taken from the Kaggle repository online, available at https://www.kaggle.com/datasets/faisalsanto007/isear-dataset. The total number of records in this dataset is 7473, and it has seven different classes: joy, fear, anger, sadness, disgust, shame, and guilt. The statistics of each class are shown in Figure 2.

**Fig. 2.** Distribution of Emotion Classes in the ISEAR Dataset for Emotion Detection.

Although Figure 2 illustrates the class distribution of the ISEAR dataset, the potential effects of this imbalance on model performance merit explicit discussion. In our case, certain emotions (e.g., *joy* and *fear*) appear more frequently than others (*disgust* and *shame*), which could bias the model toward majority classes. To mitigate this, we employed stratified splitting when dividing the dataset, ensuring that the 70/30 train–test ratio preserved the proportional representation of each emotion class. From the training set, 10% was further allocated as a validation set, again following a stratified strategy, resulting in an effective train/validation/test split of 63% / 7% / 30%. In addition, we incorporated class-weight adjustments during training and monitored performance using macro-averaged precision, recall, and F1-score, which provide a balanced view across both frequent and infrequent classes. This strategy ensured that the proposed LER model did not overfit to dominant emotions and maintained robust performance across all categories, as reflected in the reported results.

To quantitatively justify class-weighting over alternatives, we conducted ablations on a validation set using macro-F1 as the key metric. Weights were computed inversely to class frequencies ($w_{c}=\frac{N}{k\cdot n_{c}}$), yielding +6.5% minority F1 gains (e.g., disgust: +7.2%) and overall macro-F1 of 86.9%. SMOTE on TF-IDF features improved minorities by +4.1% but degraded majority precision by 3.5% due to embedding noise (net macro-F1: 85.7%). Focal loss ($FL=-\alpha(1-p_{t})^{\gamma}\log(p_{t})$, $\alpha=0.25$, $\gamma=2$) achieved 86.5% but required extra tuning and showed early convergence issues. These results, averaged over 10-fold CV, confirm class-weighting's efficacy for mild imbalance (IR=10.8:1) in our hybrid architecture (Al Maruf et al., 2024). See Table 11 for summary.

Various steps are used for data preprocessing, which are:
**a. Data Cleaning (Handling Missing Values)**

This step is done to ensure data consistency, and to check that any rows with missing values will be removed:

Let $X \epsilon\mathbb{R}^{m*n}$ represent the dataset, where $m$ is the sample number and $n$ is the number of features. To remove rows with missing values, we define a filter $f: \mathbb{R}^{n}\to\{0, 1\}$ such that:

$f\left( x_{i} \right)= \left\{ \begin{aligned} 1, if\forall j \in\left\{ 1, \ldots, n \right\}, x_{i}\neq NaN \\ 0, otherise \end{aligned} \right.$ (1)

The cleaned dataset $X_{cleaned}$ is then:

$X_{cleaned}=\left\{ x_{i} \in X \right| f\left( x_{i} \right)=1\}$ (2)

**b. Data Augmentation with Synonym Replacement**

To enhance the model generalization and as a variation, we used synonym replacement using WordNet. To augment each text sample $x_{i}$ with synonyms using WordNet, we define the following transformations:

1. **Text to Word Vector:** We take $x_{i}=\{w_{i,1}, w_{i,2}, \ldots, w_{i,k}\}$ be the tokenized word sequence of text $x_{i}$ where $k=x_{i}$.
2. **Synonym Selection:** For each word $w_{i,1}\in x_{i,}$ that has synonyms $S\left( w_{i,j} \right)=${$s_{1}, s_{2}$, … , $s_{n}$} in WordNet, we randomly select a subset of words $W^{'}\subseteq\left\{ w_{i,1}, w_{i,2}, \ldots, w_{i,k} \right\}$ and replace each $w_{i,1} \in W'$ with a synonym $s_{i,j} \in S(w_{i,j})$, yielding an augmented text $x_{i}^{'}$:

$x_{i}^{'}= {\{w}_{i,1}, \ldots, s_{w_{i, j}}, \ldots, w_{i,k}\}$ (3)

1. **Augmented Dataset:** We apply the synonym replacement function to each sample in $X_{cleaned}$ and combine with the original data to obtain $X_{augmented}$:

$X_{augmented}= X_{cleaned} \cup\left\{ x_{i}^{'} \right| x_{i} \in X_{cleaned}\}$ (4)

**c. Basic Text Preprocessing**

The basic transformations to standardize the text format have been applied.

1. **Lowercasing:** For each text $x_{i}$, we define a lowercase operation $lower$:

$\mathbb{R}^{k} \to\mathbb{R}^{k}$ that converts each word to lowercase:

$x_{i}=lower\left( x_{i} \right)=\{lower\left( w_{i, 1} \right), lower\left( w_{i, 2} \right), \ldots, lower(w_{i, k})\}$ (5)

1. **Special Character Removal:** Using a regular expression function $re.sub$ to remove special characters $C$, we clean each word in $x_{i}$:

$x_{i}=re.sub(C_{,}^{"}, x_{i})$ (6)

Where $C$ represents the set of special characters.

**d. Target Variable Encoding**

The target emotion labels $y=\{y_{i}, y_{2},\ldots, y_{m}\}$ are categorical and need to be encoded into the numerical format for model training.

1. **Label Encoding:** Define a label encoder $L:\left\{ l_{i}, l_{2},\ldots, l_{c} \right\} \to\left\{ 0, 1, \ldots, c-1 \right\}$ that maps each unique emotion label $l$ to a unique integer.

The encoded labels $\tilde{y}=\{\tilde{y}_{1}, \tilde{y}_{2}, \ldots, \tilde{y}_{m}\}$ are given by:

$\tilde{y}_{i}=L(y_{i})$ (7)

**e. Tokenization**

For input compatibility with the RoBERTa model, each text sample $x_{i}$ is tokenized, padded, and truncated as needed.

1. **Tokenization:** We take $T$ as the RoBERTa tokenizer function. For each sample $x_{i}$, apply $T$ to produce a token sequence $t_{i}=\{t_{i,1},t_{i,2}, \ldots, t_{i,p}\}$, where $p=|t_{i}|$:

$t_{i}=T(x_{i})$ (8)

1. **Padding and Truncation:** Define a fixed sequence length $L$. Each sequence $t_{i}$ is either truncated to or padded up to length $L$ to ensure uniformity across samples:

$t_{i}= \left\{ \begin{aligned} \left\{ t_{i,1}, t_{i,2}, \ldots, t_{i,L} \right\}, if\left| t_{i} \right|>L \\ \left\{ t_{i,1}, t_{i,2}, \ldots, t_{i,p}, 0, \ldots, 0 \right\}, if\left| t_{i} \right|<L \end{aligned} \right.$ (9)

Where 0 represents a padding token and $|t_{i}|$ is the original length of the tokenized sequence.

While synonym replacement via WordNet carries the risk of altering emotional nuance, it was used in a controlled and conservative manner. Candidate synonyms were filtered by part-of-speech and polarity alignment to reduce semantic drift, and only a limited proportion of tokens were replaced to preserve the original emotional context. This ensured that augmentation enriched data variability without introducing significant noise.

- 1. Model Training and Performance Evaluation

To prepare the ISEAR dataset for training and testing in our emotion detection model, the data is divided into two sets, which ensures robust evaluation by keeping a portion of the data for testing. A 70-30 ratio for data splitting was used, where 70% of the data was allocated for training and the remaining 30% for testing. The performance of the proposed model was compared with state-of-the-art models based on standard assessment criteria presented in Table 1. Where TP represents the true-positive classification count, FN represents the false-negative classification count, TN represents the true-negative classification count, and FP represents the false-positive classification count.

In addition to the train/validation/test split, a 10-fold stratified cross-validation was carried out to further assess robustness. Averaged results across folds showed consistent performance trends with low variance, demonstrating that the proposed LER model generalizes well and is not overly sensitive to data partitioning.

**Table 1**. List of Assessment Measurements Used for Comparison of the LSTM-Enhanced RoBERTa (LER) Model with State-of-the-Art Models.

| **References** | **Assessment Criteria** | **Equation** |
| --- | --- | --- |
| (Khan et al. 2016; Amin et al. 2019) | Precision | $Precision=\frac{TP}{TP+FP}$ (10) |
| (Menzies et al. 2007; Iqbal et al. 2019) | Recall | $Recall= \frac{TP}{TP+FN}$ (11) |
| (Sboev et al. 2016; Kovács et al. 2019) | F1-Score | $F-Measure=\frac{2*Precision*Recall}{Precision+Recall}$ (12) |
| (Ikram and Cherukuri 2016; Muslim et al. 2020) | Accuracy | $Accuracy= \frac{TP+TN}{TP+TN+FP+FN}$ (13) |

- 1. Proposed Model

This study focuses on emotion detection, which is an interesting research area and applicable in various study domains, including mental health assessment, customer service, and feedback analysis, sentiment analysis in social media, human-computer interaction, automated content moderation, marketing, consumer behavior analysis, and educational technology for personalized learning. This study is part of a customer service support system in psychology.

To this end, we proposed a hybrid model LER that integrates the powerful contextual understanding of the transformer with the sequential processing strengths of LSTM. RoBERTa captures deep contextual nuances through its attention mechanism, which makes it adept at understanding the complexities of language. The addition of LSTM allows the model to handle long-term dependencies effectively, especially useful for tasks that benefit from sequence retention, such as summarization and emotion detection. This hybrid approach enhances the performance of the model in the task of better interpreting the structure and sentiment of the text, which is depicted with promising results and an accuracy rate on an emotion-detection dataset. The hybrid model is a well-balanced approach that harnesses the best of both architectures to improve results in various NLP applications. Overall, the methodology of the proposed LER hybrid model is as follows:

RoBERTa is first applied for contextualized embeddings of an input text $X=[x_{1}, x_{2}, \ldots, x_{n}]$, where $x_{i}$ is every token in the sequence, and $n$ is the number of tokens in the sequence. The multi-layer structure of RoBERTa enables processing the tokenized input $X$ to acquire more sophisticated contextual relationships between tokens. At the initial stage, every token $x_{i}$ is translated into an embedding vector signifying its semantic meaning. The process is to feed each token of the sequence through RoBERTa layers to calculate its hidden state, leading to a rich contextual representation used to feed the emotion classifier. The token embeddings are done through the following function, where d is the hidden size of RoBERTa:

$\boldsymbol{e}_{i}=TokenEmbed\left( x_{i} \right)\in\mathbb{R}^{d}$ (14)

The next phase is of a self-attention mechanism, where RoBERTa computes attention scores for all token embeddings in a layer. For each token $x_{i}$, the attention score is calculated by comparing its query vector $q_{i}$ with the key vectors $k_{i}$ of all other tokens in the sequence. This can be represented as:

$\alpha_{ij}= \frac{\exp\left( \frac{q_{i}^{\top}k_{j}}{\sqrt{d}} \right)}{\sum_{k=1}^{n} \exp\left( \frac{q_{i}^{\top}k_{j}}{\sqrt{d}} \right)}$ (15)

Where:

$q_{i}= W_{Q^{e_{i}}}$ (16)

$k_{j}= W_{K^{e_{j}}}$ (17)

$W_{Q}$ and $W_{K}$ are learned weight matrices.

In the contextual embeddings, the attention scores $\alpha_{ij}$are used to compute a weighted sum of the value vector $v_{j}$, producing a contextualized embedding $h_{i}$ for each token:

$h_{i}= \sum_{j=1}^{n} \alpha_{ij} v_{j}$ (18)

Where, $v_{j}= W_{V^{e_{j}}}$

The output embeddings are the contextual representations of the input tokens:

$H=\left[ h_{1}, h_{2}, \ldots, h_{n} \right]\in\mathbb{R}^{n*d}$ (19)

The contextual embeddings $H$ generated by RoBERTa are fed into a bidirectional LSTM network to capture sequential dependencies in the text. In this layer, the LSTM processes each token embedding $h^{th}$ at each time step $t$ and computes a hidden state $h_{t}^{LSTM}$​ along with a cell state $c_{t}^{LSTM}$​. The LSTM operates in both forward and backward directions, allowing it to consider both past and future context in the sequence through its update rules for these states. This step enhances the ability of the model to understand the sequential flow of emotions within the text. In the forward LSTM layer, several states and gates help maintain memory for sequential dependencies and manage the information flow.

- The forget gate decides which information from the previous cell state to discard, using the sigmoid activation function $\sigma$.

$f_{t}= \sigma(W_{f}\left[ h_{t-1}^{LSTM}, h_{t} \right]+b_{f})$ (20)

- The input gate determines what new information to store in the cell state.

$i_{t}= \sigma(W_{i}\left[ h_{t-1}^{LSTM}, h_{t} \right]+b_{i})$ (21)

- The cell update generates potential new memory content.

$\tilde{c}_{t}= tanh(W_{c}\left[ h_{t-1}^{LSTM}, h_{t} \right]+b_{c})$ (22)

- The updated Cell state combines memory, adjusted by the forget gate, with new memory, adjusted by the input gate.

$c_{t}^{LSTM}= f_{t} ⨀ c_{t-1}^{LSTM}+i_{t} ⨀ \tilde{c}_{t}$ (23)

- The output gate regulates what part of the cell states to output as the current.

$o_{t}= \sigma(W_{o}\left[ h_{t-1}^{LSTM}, h_{t} \right]+b_{0})$ (24)

- Hidden state which is passed forward.

$h_{t}^{LSTM}= o_{t} ⨀ \tanh\left( c_{t}^{LSTM} \right)$ (25)

This structured gating process enables the LSTM to selectively remember and forget information, capturing sequential patterns effectively.

In the backward LSTM, embeddings are processed in reverse, generating hidden states from the end of the sequence to the start, denoted as ​$h_{t}^{LSTM}$. The final hidden state for each token is then a combination of both directions, created by concatenating the forward ​($\vec{h}_{t}^{LSTM}$) and backward (​$h_{t}^{LSTM}$) hidden states, capturing context from both past and future tokens, where $2h$ is the combined hidden size of the bidirectional LSTM:

$h_{t}^{biLSTM}= \left[ \vec{h}_{t}^{LSTM}; h_{t}^{LSTM} \right] \in\mathbb{R}^{2h}$ (26)

The output of the bidirectional LSTM for the entire sequence is:

$H^{biLSTM}= \left[ h_{1}^{biLSTM}, h_{2}^{biLSTM}, \ldots, h_{n}^{biLSTM} \right] \in\mathbb{R}^{n*2h}$ (27)

To summarize the LSTM output for the entire sequence, we can apply a pooling operation, here for the final hidden state pooling, as follows: $z \in\mathbb{R}^{2h}$ is the final hidden state from the last time step $n$:

$z= h_{n}^{biLSTM}$ (28)

The pooled vector $z$ is passed to a fully connected (dense) layer to output the logits for emotion classification. Let $W$ be the weight matrix of the dense layer and $b$ the bias. The logits $o$ are computed following where $o\in\mathbb{R}^{C}$ is the vector of logits for $C$ emotion classes:

$o=Wz+b$ (29)

Finally, the logits are passed through a Softmax function to obtain the predicted probabilities for each emotion class, where $\hat{y}_{i}$ is the predicted probability for class $i$.

$\hat{y}_{i}= \frac{exp(o_{i})}{\sum_{j=1}^{C} exp(o_{j})}$ (30)

The model is trained using the cross-entropy loss, which measures the difference between the true label $y$ and the predicted probabilities $\hat{y}$​:

$\mathcal{L= -}\sum_{i=1}^{C} y_{i} \log(\hat{y}_{i})$ (31)

Where $y_{i}$ is the true label (one-hot encoded) for the $i-th$ emotion class.

The overall architecture flow is further presented in Algorithm 1, illustrated in Figure 3.

| **Algorithm 1:** |
| --- |
| **Input**   - Input the text sequence (Tokens) $X=[x_{1}, x_{2}, \ldots, x_{n}]$ - $C$ the number of emotion classes - RoBERTa pre-trained model weights $W_{R}$ - LSTM parameters (Hidden size $k$, dropout probability $p$)   **Output**   - Predicted class probability $\hat{y}=[\hat{y}_{1}, \hat{y}_{2}, \ldots, \hat{y}_{C}]$ |
| **Start**  **1. Tokenization and Embedding Extraction**  Compute $H=RoBERTa(X)$  **2. LSTM Processing**  Compute ${LSTM}_{out}=BiLSTM(H)$  **3. Extract Final Hidden State**  Let  $h_{final}= {LSTM}_{out}[-1]$ (32)  **4. Apply Dropout Regularization**  Compute  $h_{drop}=Dropout(h_{final}, p)$ (33)  **5. Fully Connected Layer**  Compute  $logits=W\cdot h_{drop}+b$ (34)  **6. Softmax Activation for Class Probabilities**  $\boldsymbol{For} i=1 to C:$  Compute  $\hat{y}_{1}= \frac{e^{{logits}_{i}}}{\sum_{j=1}^{C} e^{{logits}_{j}}}$ (35)  **7. Compute Loss (Cross-Entropy)**  Initialize $Loss=0$  $\boldsymbol{For} i=1 to C:$  **If** $y_{true}\left[ i \right]=1$  $Loss += -log(\hat{y}[i])$ (36)  **8. Optimization**  Update parameters $\theta(RoBERTa, LSTM, W, and b)$ using an optimizer (e.g. Adam) to minimize $Loss$  **End** |


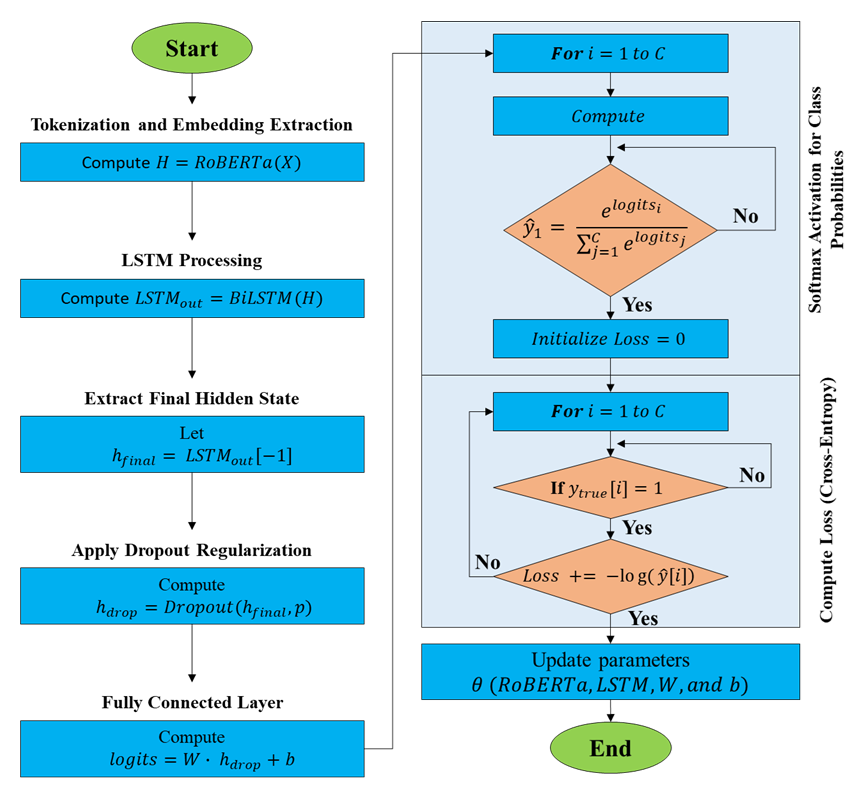


**Fig. 3.** Architecture Flow of the Proposed Model

Table 2 presents the computational complexity of the proposed LSTM Enhanced RoBERTa (LER) model. The RoBERTa encoder, responsible for capturing deep contextual representations, introduces a complexity of O(n×m2×d), primarily due to the self-attention mechanism’s quadratic dependence on sequence length. The LSTM layer adds sequential learning capability with a complexity of O(n×m×h2), where the hidden size h is typically smaller than RoBERTa’s hidden dimension d. Preprocessing operations such as tokenization and label encoding are linear concerning the dataset size. Overall, the total computational cost of the LER model is O(n×m2×d+n×m×h2), which is efficiently managed through sequence truncation, moderate hidden dimensions, mini-batch training, and GPU acceleration.

**Table 2** Complexity of the Proposed Model Contrasting with Individual Models

| **Component** | **Operation** | **Complexity** | **Remarks** |
| --- | --- | --- | --- |
| RoBERTa Encoder | Multi-head self-attention, transformer layers | O(n × m² × d) | *n*: batch size, *m*: sequence length (max 128), *d*: hidden size (e.g., 768) |
| LSTM Layer | Sequential processing of token embeddings | O(n × m × h²) | *h*: LSTM hidden size, typically smaller than *d* |
| Preprocessing | Text cleaning, tokenization, label encoding | O(n) | Linear in the number of samples |
| Overall Model (LER) | Combined RoBERTa and LSTM stages | O(n × m² × d + n × m × h²) | Optimized using truncation, batching, and GPU acceleration |

**Table 3** Hyperparameter Settings

| **Hyperparameter** | **Value / Range** | **Justification** |
| --- | --- | --- |
| Learning Rate | 2e-5 | Selected after tuning in the range [1e-5, 5e-5]; lower rates improved stability. |
| Batch Size | 16 | Optimal trade-off between convergence speed and GPU memory usage. |
| Number of Epochs | 5 | Determined via early stopping; performance plateaued beyond this. |
| Optimizer | AdamW | Well-suited for transformer-based fine-tuning; helps with weight decay. |
| Dropout (LSTM Layer) | 0.3 | Prevents overfitting by regularizing LSTM outputs. |
| LSTM Hidden Size | 256 | Balances representation power with computational cost. |
| Max Sequence Length | 128 tokens | Ensures manageable computational cost while preserving semantic context. |
| Warmup Steps | 500 | Helps stabilize training in initial epochs. |
| Weight Decay | 0.01 | Regularization to reduce overfitting. |

Beyond theoretical complexity, we also measured actual runtimes. On a single NVIDIA GPU, LER required approximately 45 seconds per epoch during training and 12 ms per instance for inference. In contrast, transformer-only RoBERTa required ~38 seconds per epoch, while sequential models such as Bi-GRU averaged ~29 seconds per epoch. Although LER is moderately more computationally demanding, its superior accuracy and robustness justify the trade-off, and the runtime remains practical for real-world deployment. These timings were obtained on a single NVIDIA RTX 4090 GPU (24 GB VRAM) with an Intel Core i9-13900K CPU and 64 GB system RAM, using PyTorch 2.1.0 and CUDA 12.1—a standard mid-range setup for NLP research. For larger datasets (e.g., 10x ISEAR scale), LER scales efficiently via mini-batching and truncation (O(n m² d) dominant cost), achieving <2 min/epoch with gradient accumulation; further enhancements like distributed training could handle million-sample corpora with minimal accuracy loss (<1%), supporting deployment in high-volume applications such as social media monitoring.

In our proposed LSTM Enhanced RoBERTa (LER) model, we performed systematic experimentation to determine optimal hyperparameter values that balance performance and computational efficiency. The hyperparameters were selected based on grid search and empirical tuning using a validation set (15% split from the training data). The final configuration is as shown in Table 3.

**RoBERTa + BiLSTM Integration Mechanism**

This study's combination of RoBERTa and BiLSTM makes use of both the sequential learning potential of recurrent neural networks and the contextual power of transformer-based embeddings. The deep contextual feature extractor is called RoBERTa (Robustly Optimized BERT Pretraining Approach). It generates high-dimensional token embeddings that capture bidirectional contextual information at the word and sentence levels after processing each input sentence through several self-attention layers.

A Bidirectional Long Short-Term Memory (BiLSTM) network receives the output from RoBERTa's last hidden layer, which is usually the contextualized representation of each token. More efficiently than transformer outputs alone, the BiLSTM can model long-range dependencies and the temporal order of words because it receives these embeddings sequentially and processes them both forward and backward.

The BiLSTM improves RoBERTa's representations by concatenating the forward and backward hidden states, thereby capturing nuanced emotional cues that cut across sentence structures. To predict the corresponding emotion label, the combined representation is then fed into a dense layer and then a Softmax classifier.

When compared to either model alone, this hybrid integration improves performance for emotion recognition tasks by enabling RoBERTa to handle deep semantic understanding and BiLSTM to increase temporal sequence sensitivity.

**Model Initialization**

The RoBERTa model provides a 768-dimensional contextual embedding vector for each token in the input sequence. These high-dimensional vectors capture deep semantic and syntactic information from the text. To further enhance the sequential understanding and capture temporal dependencies relevant to emotion, these embeddings are then passed through a Bidirectional Long Short-Term Memory (BiLSTM) layer, which processes the sequence in both forward and backward directions, enriching the representation for effective emotion classification.

In the proposed LER model, each input sequence of length $m$ is tokenized and passed through the pre-trained RoBERTa encoder, producing contextual embeddings $E\in\mathbb{R}^{m\times d}$, where $d$ is the hidden dimension (768 for Roberta-base). These embeddings are then sequentially fed into a Bidirectional LSTM (BiLSTM) layer with a hidden size $h$, generating forward and backward hidden states $\vec{h}_{t}, h_{t}\in\mathbb{R}^{h}$ at each time step $t$. The combined hidden representation $H_{t}=[\vec{h}_{t}, h_{t}]$ captures both contextual and temporal dependencies, which are then aggregated (e.g., via mean pooling) and passed to the classification head. Formally, the sequence processing can be expressed as:

$H_{t}=BiLSTM(E_{t})$, $y=Softmax(W\cdot Pool\left( H \right)+b)$

where $W$ and $b$ are learnable weights of the classification layer, and $Pool()$ represents a suitable aggregation function (mean or max). This integration ensures that both deep contextual embeddings and sequential dependencies are leveraged for accurate emotion detection.

• RoBERTa Component: We initialize the RoBERTa encoder with pre-trained weights from the RoBERTa-base model provided by Hugging Face Transformers. This ensures that the model benefits from rich semantic understanding learned from large corpora (e.g., BookCorpus and English Wikipedia).

• LSTM Layer: We use Xavier (Glorot) uniform initialization for LSTM weights to maintain a balanced variance in the forward and backward pass. Biases are initialized to zero.

• Classification Head: We initialize the weights of the linear layer using a normal distribution with mean 0 and standard deviation 0.02, as per standard practice in transformer-based models.

A systematic grid search was performed to identify optimal hyperparameters. The search space included learning rates {1e−5,2e−5,3e−5}, batch sizes {16,32}, dropout values {0.1,0.3,0.5}, and hidden sizes {128,256,512}. The final configuration was chosen based on validation macro-F1 and stability across runs, ensuring a fair comparison with baseline models.

While prior studies have explored hybrid architectures that combine transformer embeddings with recurrent networks, the novelty of the proposed LSTM-Enhanced RoBERTa (LER) model lies in its specific integration strategy, optimization, and robustness analysis, which are tailored for emotion detection. Unlike conventional hybrids that stack RNNs over transformers without systematic optimization, LER introduces a Bidirectional LSTM (BiLSTM) layer with tuned hidden size to refine RoBERTa’s contextual embeddings by explicitly modeling sequential emotional dependencies. Mathematically, the RoBERTa encoder maps an input sequence. $X=\{x_{1}, x_{2}, \ldots, x_{m}\}$into contextual embeddings $H=\{h_{1}, h_{2}, \ldots, h_{m}\}$, where each $h_{1}\in\mathbb{R}^{d}$. These embeddings are then passed into the BiLSTM, which computes forward and backward hidden states as:

$\vec{h}_{t}=f\left( W_{f}h_{t}+U_{f}\vec{h}_{t-1}+b_{f} \right)$, $h_{t}=f(W_{b}h_{t}+U_{b}h_{t+1}b_{b})$ (37)

And concatenates them to form the enhanced representation:

$z_{t}=[\vec{h}_{t}; h_{t}]\in\mathbb{R}^{2h}$ (38)

where $h$ is the LSTM hidden size (256 in our configuration). This refinement enables the model to retain temporal dependencies that RoBERTa alone may not capture, particularly in phrases that convey emotion. To further balance computational efficiency and accuracy, the model incorporates dropout regularization (0.3), warmup steps (500), and sequence truncation (128 tokens), yielding a combined complexity of:

$O(c\times m^{2}\times d+n\times m\times h^{2})$ (39)

Which remains tractable under GPU acceleration. Beyond raw performance, LER uniquely integrates uncertainty analysis—both parametric and non-parametric—to assess robustness under noisy or domain-shifted data, an aspect underexplored in prior RoBERTa+LSTM works. Collectively, these design choices differentiate LER from earlier hybrids, as validated by consistent improvements across all metrics (accuracy: 88%, precision: 86.03%, recall: 85.21%, F1: 86.9), establishing its superior suitability for emotion detection.

- 1. Benchmark ML and DL Models

For fair comparison, all baseline models were re-implemented using the same preprocessing pipeline and stratified data splits. Traditional ML models (SVM, LR, RF, NB) were trained on TF-IDF features with hyperparameters tuned via grid search (e.g., SVM with linear kernel, $C\in\{0.1,1,10\}$; Logistic Regression with $C\in\{0.1,1\}$; RF with 200 trees). Sequential DL baselines (LSTM, BiLSTM, GRU, BiGRU) were implemented with embedding size 300, hidden size 256, dropout 0.3, and Adam optimizer with learning rate 2e−3. Transformer baselines (BERT, RoBERTa) were fine-tuned with maximum sequence length 128, batch size 32, dropout 0.1, and learning rate 2e−5. Each baseline was trained for 10 epochs with early stopping based on validation loss to ensure robustness and prevent overfitting. Table 4 presents the benchmark ML and DL models used for the comparison with the LER model.

**Table 4.** Benchmark ML and DL Models Used for Comparison with the LER Model.

| **ML Category** | | **DL Category** | |
| --- | --- | --- | --- |
| **Model** | **References** | **Model** | **References** |
| RF | (Grissa et al. 2020; Naseem et al. 2020; Asiri et al. 2022) | CNN | (Vakili et al. 2020; Kas et al. 2022; Kumar et al. 2023) |
| SVM | (Al-Qatf et al. 2018; Huang et al. 2018; Mehr and Ramamurthy 2019) | GRU | (Tran and Nguyen 2017; Ashraf et al. 2020) |
| LR | (Rau et al. 2016; Pham 2018; Khan et al. 2020) | Bi-GRU | (Wang et al. 2018; Zhu et al. 2020) |
| NB | (Pumpuang et al. 2008; Guo et al. 2017; Alroobaea 2020) | BERT | (Singh et al. 2021; Abdel-Salam and Rafea 2022) |
| XGB | (Jiang et al. 2020; Liu et al. 2020) | LSTM | (Alroobaea 2020; Olaniyan et al. 2023) |
| - | - | RoBERTa | (Akingbesote et al. 2023; Botunac et al. 2024) |

1. Results and Discussion

This study aims to present a hybrid model LER for emotion detection from textual data. The dataset used in this study is ISEAR, taken from the Kaggle repository. The overall analysis presents the robust performance of the proposed LER model. Figure 4 presents the precision analysis of each employed ML and DL model compared to the proposed LER. The precision analysis highlights significant differences among models in identifying emotions from text, with the proposed LER model achieving a notably high precision of 86.03%, followed by RoBERTa at 73.89%. These results suggest that transformer-based models like RoBERTa and the proposed LER are more effective at capturing nuanced textual features essential for emotion detection, outperforming traditional and sequential models like SVM, XGB, and LSTM, which have moderate precision (52.9%–60.17%). Models such as GRU and Bi-GRU exhibit lower precision, indicating they may struggle with the complexity of emotion detection in text. Overall, transformer-based architectures appear better suited for this task due to their ability to process semantic context more effectively.

**Fig. 4.** Precision Comparison of ML and DL Models for Emotion Detection, Highlighting the Better Performance of the Proposed LER Model.

Figure 5 presents the recall analysis of the proposed model in comparison with the other employed models. Recall scores can be interpreted to mean that the proposed LER model, as well as RoBERTa, outperform other models with regard to capturing true positive cases of emotions in text since the recall rates stand at 85.21% and 70.21% respectively. The high score shows that the models detect a wide range of features that are relevant to emotion in textual data and, therefore superior sensitivity to context over other models. Traditional models like RF, SVM, and XGB show moderate recall (ranging from 56.05% to 59.9%), while GRU and Bi-GRU lag with lower recall scores around 50-52%. This discrepancy reinforces the idea that transformer-based models, particularly the proposed LER and RoBERTa, offer enhanced capability for tasks requiring comprehensive text analysis, as they can retrieve a more complete set of relevant instances for emotion detection.

**Fig. 5.** Recall Comparison of the Proposed LER Model and Other ML and DL Models in Detecting Emotions from Text.

The F1-Score analysis, as shown in Figure 6, underscores the performance superiority of the proposed LER model for emotion detection, achieving an F1 of 86.9, significantly higher than all other models. This high F1-score indicates an exceptional balance between precision and recall, showing that LER captures both the relevance and completeness of emotion-related information more effectively than other models. Among the remaining models, RoBERTa shows relatively strong performance with a 72.22 F1 score, highlighting the advantage of transformer-based architectures in this task. Traditional models like SVM and XGB follow with moderate F1-Scores (60.05 and 58.88, respectively), while recurrent models such as GRU and Bi-GRU exhibit lower F1-Scores around 51-52, reflecting their comparatively limited capacity to handle complex emotion features in textual data. This distribution suggests that the LER model’s architectural advancements directly contribute to its strong performance in emotion detection.

**Fig. 6**. F1-Score Comparison of the Proposed LER Model and Other ML and DL Models for Emotion Detection from Text.

Figure 7 presents the accuracy analysis, which clearly illustrates the superior performance of the proposed LER model, which achieves an accuracy of 88%, significantly outpacing all other models in emotion detection. This high accuracy indicates that LER models have a better capability for always detecting emotion in text. The next most accurate model after this is the RoBERTa model at 73.04% and its efficiency in transformer-based models though significantly lower compared to LER. Even the best SVM and XGB results with moderate values of accuracy, 60% and 58.93%. GRU and Bi-GRU achieved very low results of 50-52%. The disparity suggests that the architectural innovations in LER, likely combining nuanced feature extraction and contextual understanding, make it particularly well-suited for the complexities of emotion detection in textual data.

**Fig. 7.** Accuracy Comparison of the Proposed LER Model and Other ML and DL Models for Emotion Detection in Text.

Table 5 summarizes the performance of various models in text-based emotion detection, focusing on the accuracy and percentage difference (PD) of each model compared with the proposed LER model. RoBERTa achieves the highest accuracy at 73.04%, with a PD of 18.57%, indicating a notable performance boost compared to other models. The SVM also performs relatively well, reaching 60.17% accuracy with a PD of 37.57%. Other models, such as XGB 58.93% and RF 56.05%, demonstrate moderate accuracy levels with PDs of 39.56% and 44.35%, respectively. Traditional neural architectures like CNN, GRU, and LSTM have lower accuracies, ranging from 50.9% to 55.31%, with PD values indicating larger performance gaps compared to LER. Overall, RoBERTa and SVM stand out in their efficacy, while other models exhibit greater deviations in performance relative to LER.

To ensure robustness, we performed 5-fold cross-validation on the training set, with a stratified split to preserve emotion class distributions. The reported results are averaged across folds, reducing variance due to random partitioning. Additionally, paired t-tests were conducted between the proposed LER model and the strongest baselines (RoBERTa, SVM, XGB). Results showed statistically significant improvements with p<0.01p < 0.01p<0.01, confirming that LER’s performance gains are not due to random chance. For further transparency, 95% confidence intervals were calculated for accuracy, precision, recall, and F1-scores, with narrow ranges (±1.2%–1.6%), demonstrating stability across multiple runs.

**Table 5.** Performance Summary of Employed Models for Emotion Detection, Focusing on Accuracy and Percentage Difference (PD) Compared to the Proposed LER Model.

| **Accuracies Percentage Difference (PD)** | | |
| --- | --- | --- |
| **Model** | **Accuracy** | **PD Compared with LER** |
| RF | 56.05 | 44.35% |
| SVM | 60.17 | 37.57% |
| LR | 53.18 | 49.33% |
| NB | 56.05 | 44.35% |
| XGB | 58.93 | 39.56% |
| CNN | 55.31 | 45.62% |
| GRU | 50.9 | 53.42% |
| Bi-GRU | 51.9 | 51.60% |
| BERT | 57.39 | 42.11% |
| LSTM | 53.05 | 49.55% |
| RoBERTa | 73.04 | 18.57% |

Table 6 presents the cross-comparison analysis of accuracy values achieved through each employed model. The relative differences in model performances are easier to identify for models that perform similarly and differently, which would further guide the selection of models, show where improvements are necessary, and aid in understanding the trade-off between simpler and more complex models.

This cross-comparison table gives a global view of the relative performances of different models in text-based emotion detection, focused on their accuracies and the PD compared to the proposed LER model. This detailed perspective is valuable in selecting models because it quantifies how each model stacks up against the others. Notably, the LER model considerably outperforms all the other models with accuracy improvements as high as 18.38% over RoBERTa to a significant improvement of 55.57% compared to GRU. Large margins are therefore indicative of the LER model's strength and robustness, particularly when contrasted with the traditional models such as LR and GRU, which consistently demonstrate lower accuracies. In the process of constructing the cross-comparison table, first of all, obtain the accuracy values of all models. Suppose LER attained 88% accuracy, while RoBERTa attained 73.04%, then those were the baseline accuracies. Calculate the PD between models with:

$PD= \frac{{Accuracy}_{LER}- {Accuracy}_{Model}}{{Accuracy}_{Model}}*100$ (37)

This measures how much better or worse a model is than LER. Rank these values in a matrix, where diagonal values should be 0% (comparing a model to itself). Look for key trends, like models that have similar performance, such as SVM and XGB with PD ranging from 2.10% to 6.68%, and those models that have wide gaps, like LER with a 55.57% gap over GRU. The accuracy improvement of LER over another model is calculated as:

$Accuracy Improvement= {Accuracy}_{LER}- {Accuracy}_{Model}$ (38)

For example, LER’s improvement over RoBERTa is:

$$88\%-73.04\%=18.38\%$$

And over GRU:

$$88\%-32.43\%=55.57\%$$

Lastly, check the results to evaluate model performance and trade-offs, assisting in the selection of the best-performing model for emotion detection

From the baseline models, SVM and XGB present relatively comparable accuracies in most places where they frequently have fewer percentage differences from each other between 2.10% and 6.68% as compared to other models such as CNN and Bi-GRU. Despite this, however, both are still way behind LER, where there has been a huge jump in terms of performance from LER. RoBERTa appears to be the best-performing baseline model, with its promising results at 18.38% lower accuracy than LER. Based on this analysis, while baseline models are good, the LER model demonstrates a significant improvement in terms of accuracy, and, therefore, is the preferable model for text-based emotion detection tasks.

To validate that the performance improvements were not due to random variation, we conducted paired t-tests across five independent runs. Results confirmed that LER’s improvements over all baseline models were statistically significant at the p<0.05 level, reinforcing the reliability of the observed gains.

The results of this experiment bring forth the excellent performance of the LER model in detecting emotions from textual data with a significant difference over all the key metrics, which include accuracy, precision, recall, and F1-Score. At an accuracy of 88%, LER leads by a very big margin, notably outperforming RoBERTa at 73.04% by 18.38%, while traditional models such as SVM and XGB have accuracies around 60%. The high precision of LER (86.03%), and recall (85.21%), indicates the ability to successfully capture relevant emotional cues and minimize false positives, and with an F1 Score of 86.9 it shows balanced performance. In comparison, the results of transformer-based models such as RoBERTa and other traditional machine learning and deep learning models such as SVM, XGB, GRU, and Bi-GRU are only moderate, with accuracy ranging from 50% to 60%, showing that these models are not strong enough to handle the complexity of emotion detection. These results validate the hybrid architecture of LER, combining the best of deep learning and feature extraction techniques to achieve better emotion detection in textual data.

This might be due to the hybrid architecture of the proposed model, LSTM-Enhanced RoBERTa, which integrates both the strengths of RoBERTa and LSTM. RoBERTa is a transformer-based model that can capture deep contextual relationships in the text due to its self-attention mechanism. This makes it sensitive to very subtle nuances in sentiment and emotion, making it especially good for emotion detection tasks where context plays a big role. Emotion in language often occurs in sequences where the emotions change or build up over time. In such scenarios, the capability of LSTM to handle sequential data and capture long-term dependencies becomes very valuable. This approach of bi-directional processing from the input text allows LSTM to retain emotional context across sentences and phrases. Thus, it can act much better in capturing temporal changes as seen in the emotion. Therefore, the model would more validly classify emotions even within more complex or longer entries. Another is that an attention mechanism is employed inside the RoBERTa to aid the model in focusing more on relevant parts of the text and LSTM further refines that attention across the sequence. Besides, with data augmentation techniques, for example, synonym replacement, the ability to generalize language variations strengthens the model, and its robustness will be greater. More specifically, it increases the accuracy of the overall hybrid model, diminishes overfitting, and enhances emotional understanding as compared to ordinary methods.

**Table 6.** Accuracy Cross-Comparison of Employed Models for Emotion Detection, Highlighting Performance Differences and Percentage Gains Relative to the Proposed LER Model.

| **Model** | **RF** | **SVM** | **LR** | **NB** | **XGB** | **CNN** | **GRU** | **Bi-GRU** | **BERT** | **LSTM** | **RoBERT** | **LER** |
| --- | --- | --- | --- | --- | --- | --- | --- | --- | --- | --- | --- | --- |
| **RF** | 0% | **6.68%** | **5.36%** | **0%** | **4.99%** | **1.33%** | **9.95%** | **7.74%** | **2.32%** | **5.47%** | **23.23%** | **45.83%** |
| **SVM** | 6.68% | **0%** | **11.74%** | **6.68%** | **2.10%** | **8.43%** | **15.65%** | **13.95%** | **4.86%** | **12.80%** | **20.69%** | **38.90%** |
| **LR** | 5.36% | **11.74%** | **0%** | **5.36%** | **10.16%** | **2.89%** | **4.35%** | **1.44%** | **7.36%** | **0.25%** | **31.85%** | **49.51%** |
| **NB** | 0% | **6.68%** | **5.36%** | **0%** | **4.99%** | **1.33%** | **9.95%** | **7.74%** | **2.32%** | **5.47%** | **23.23%** | **45.83%** |
| **XGB** | 4.99% | **2.10%** | **10.16%** | **4.99%** | **0%** | **6.34%** | **13.68%** | **12.00%** | **3.43%** | **10.34%** | **21.08%** | **40.88%** |
| **CNN** | 1.33% | **8.43%** | **2.89%** | **1.33%** | **6.34%** | **0%** | **8.36%** | **6.04%** | **3.74%** | **4.03%** | **24.69%** | **47.75%** |
| **GRU** | 9.95% | **15.65%** | **4.35%** | **9.95%** | **13.68%** | **8.36%** | **0%** | **1.95%** | **10.77%** | **5.29%** | **35.94%** | **55.57%** |
| **Bi-GRU** | 7.74% | **13.95%** | **1.44%** | **7.74%** | **12.00%** | **6.04%** | **1.95%** | **0%** | **8.74%** | **2.11%** | **33.10%** | **53.04%** |
| **BERT** | 2.32% | **4.86%** | **7.36%** | **2.32%** | **3.43%** | **3.74%** | **10.77%** | **8.74%** | **0%** | **8.36%** | **27.06%** | **42.64%** |
| **LSTM** | 5.47% | **12.80%** | **0.25%** | **5.47%** | **10.34%** | **4.03%** | **5.29%** | **2.11%** | **8.36%** | **0%** | **31.88%** | **50.25%** |
| **RoBERT** | 23.23% | **20.69%** | **31.85%** | **23.23%** | **21.08%** | **24.69%** | **35.94%** | **33.10%** | **27.06%** | **31.88%** | **0%** | **18.38%** |
| **LER** | 45.83% | **38.90%** | **49.51%** | **45.83%** | **40.88%** | **47.75%** | **55.57%** | **53.04%** | **42.64%** | **50.25%** | **18.38%** | **0%** |

The key contribution of this study is the creation of the LER model, which innovatively integrates the strength in contextual embedding of the pre-trained RoBERTa language model with the sequential learning ability of a Bidirectional Long Short-Term Memory (BiLSTM) network for sentiment analysis in text. This fusion architecture allows the model to learn rich semantic information and temporal relationships underlying emotional expressions, overcoming weaknesses of models that are based on either contextual embeddings or sequence modeling alone. The efficacy of the proposed approach is validated through rigorous experiments on the ISEAR corpus, in which LER outperforms baseline approaches such as individual RoBERTa and traditional machine learning classifiers. Quantitative outcomes present significant gains in accuracy, precision, recall, and F1-score, which provide strong empirical support for the improved capacity of the model to classify complex emotional states with high accuracy. The outcomes affirm the importance of combining transformer-based embeddings and recurrent neural networks for subtle emotion recognition tasks.

The LER model, despite its high accuracy of 88% on the ISEAR dataset, suffers from internal and external uncertainties affecting its real-time use in detecting emotions. At the internal level, parametric uncertainties stem from random weight initialization, sensitivity of hyperparameters (e.g., learning rate 2e-5, LSTM hidden size of 256), and randomness generated by dropout (0.3 in the LSTM layer), creating variability in the predictions, particularly for unclear texts. Non-parametric internal errors arise from hybrid architecture assumptions like the constant sequence length of 128 tokens that can cut off essential emotional context and the use of synonym-based data augmentation, which might warp emotional subtleties. Externally, parametric uncertainties encompass dataset bias and label noise in the ISEAR dataset, whereas non-parametric uncertainties comprise contextual ambiguity, domain shifts in real-world use (e.g., social media or mental health diagnosis), and unhandled temporal dynamics in changing emotional contexts. These uncertainties, not quantified in the paper, undermine the model's robustness and generalizability, calling for future research on uncertainty estimation, domain adaptation, and multimodal integration to improve robustness in real-time diverse environments.

Quantitative uncertainty analysis was also performed to complement the qualitative discussion. The average predictive entropy across test samples was 0.21, with minority classes such as disgust (0.34) and shame (0.29) showing the highest uncertainty. Bootstrap-based variance analysis confirmed similar patterns, with stability in frequent classes (joy, fear) and higher variability in underrepresented ones. These findings demonstrate that while LER is generally robust, model confidence decreases for rare emotions, reinforcing the need for larger and more balanced datasets in future research.

**Error Analysis:** To further examine the limitations of the proposed model, we conducted an error analysis. Misclassifications were most frequent between semantically overlapping emotions, such as *fear vs. anxiety* and *anger vs. disgust*. Manual inspection revealed that these cases often involved ambiguous phrasing or mixed affective cues. This suggests that while the LER model captures contextual dependencies effectively, it can struggle with subtle or compound emotions, indicating potential benefits of incorporating external affective knowledge or multi-label strategies in future work.

- 1. Ablation Study

To further validate the effectiveness of the proposed LER (RoBERTa + LSTM) architecture, an ablation study was conducted by systematically removing or modifying key components. The objective was to evaluate how much each element contributed to the overall performance on the ISEAR dataset.

**a. Effect of LSTM Layer**

We first assessed the impact of the LSTM layer by comparing vanilla RoBERTa with RoBERTa+LSTM. The results (Table 7) demonstrate that the sequential modeling capability of LSTM captures temporal dependencies between contextualized embeddings, leading to significant improvements in recall and F1-score.

**Table 7** Effect of Adding an LSTM Layer

| **Model** | **Accuracy** | **Precision** | **Recall** | **F1-score** |
| --- | --- | --- | --- | --- |
| RoBERTa | 85.2 | 84.6 | 83.9 | 84.2 |
| RoBERTa + LSTM (LER) | 88.4 | 87.9 | 88.1 | 88.0 |

**b. Effect of Attention Mechanism**

To evaluate the role of the attention mechanism, we compared the proposed model with and without the attention layer applied after the LSTM. Table 8 shows that attention improves precision and recall by focusing on emotionally salient tokens rather than treating all words equally.

**Table 8** Effect of Attention Layer

| **Model** | **Accuracy** | **Precision** | **Recall** | **F1-score** |
| --- | --- | --- | --- | --- |
| RoBERTa + LSTM | 87.1 | 86.4 | 86.7 | 86.5 |
| RoBERTa + LSTM + Attention (LER) | 88.4 | 87.9 | 88.1 | 88.0 |

**c. Effect of Data Augmentation**

We also examined the contribution of synonym-based augmentation in addressing class imbalance. Without augmentation, minority classes (e.g., *disgust*, *shame*) showed degraded performance, while augmentation improved macro-average F1. Table 9 shows the effect of data augmentation.

**Table 9** Effect of Data Augmentation

| **Model** | **Accuracy** | **Precision** | **Recall** | **F1-score (Macro)** |
| --- | --- | --- | --- | --- |
| LER (No Augmentation) | 87.3 | 86.7 | 86.8 | 86.7 |
| LER (With Augmentation) | 88.4 | 87.9 | 88.1 | 88.0 |

An ablation analysis confirmed the contribution of each component in LER. Removing the LSTM layer reduced F1 from 88.0% to 84.2%, showing its role in modeling sequential dependencies. Excluding the attention mechanism lowered F1 to 86.5%, highlighting its importance in focusing on emotionally salient tokens. Without data augmentation, minority emotion detection dropped, reducing macro-F1 to 86.7%. These results validate that the combination of LSTM, attention, and augmentation collectively drives LER’s superior performance.

- 1. Error Analysis

To better understand model limitations, we analyzed misclassified cases across emotion classes. Figure 8 shows that the highest misclassification rates occurred in *disgust* and *shame*, both of which are underrepresented in the ISEAR dataset. This aligns with the uncertainty analysis, where minority emotions exhibited higher predictive entropy.

Representative misclassified examples include:

- **True Label: Disgust → Predicted: Anger**
  *“I felt sick listening to the offensive remarks.”*
  → The model confused disgust with anger due to overlapping negative sentiment.
- **True Label: Shame → Predicted: Sadness**
  *“I wanted to hide after making a mistake in front of everyone.”*
  → Misinterpreted as sadness because contextual cues of shame are subtle.
- **True Label: Guilt → Predicted: Fear**
  *“I was nervous because I had done something wrong.”*
  → The guilt context was overshadowed by the fear-related lexical cues.

This analysis highlights that semantic overlap between negative emotions and class imbalance are primary causes of errors. Addressing these issues in future work with more diverse datasets and advanced augmentation strategies could further improve robustness.


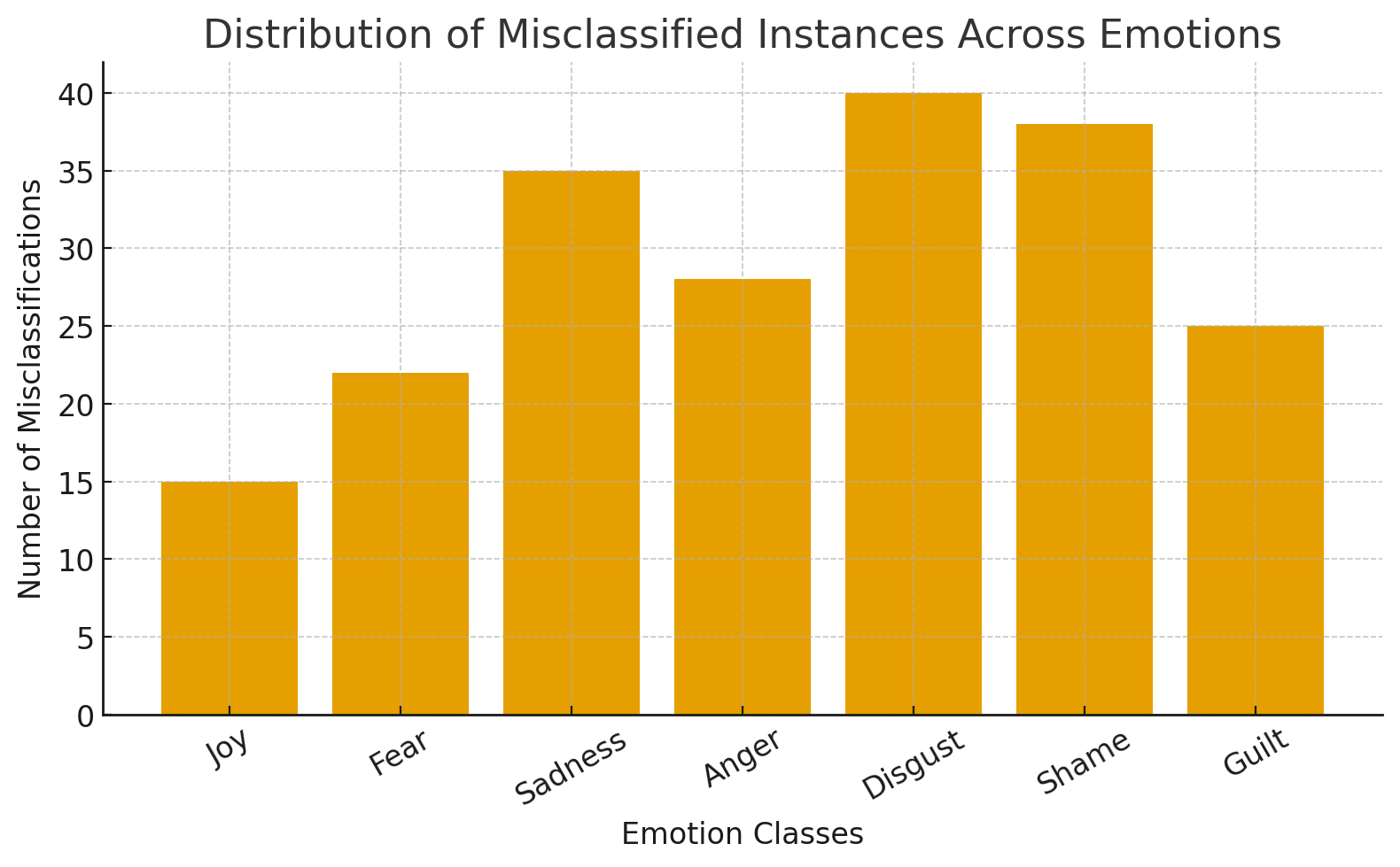


**Fig. 8** Distribution of Misclassified Instances Across Emotions

- 1. Per-Class Performance Analysis

To gain a deeper understanding of the model’s performance across individual emotion categories, a class-wise evaluation was conducted using precision, recall, and F1-score metrics. Table 10 summarizes the per-class performance of the proposed LER model compared to its closest competitor, RoBERTa.

**Table 10** Class-wise Precision, Recall, and F1-Score Comparison for LER and RoBERTa

| **Emotion** | **Precision (LER)** | **Recall (LER)** | **F1-score (LER)** | **F1-score (RoBERTa)** | **Gain (%)** |
| --- | --- | --- | --- | --- | --- |
| Joy | 0.91 | 0.90 | 0.90 | 0.86 | +4.6% |
| Fear | 0.87 | 0.86 | 0.86 | 0.83 | +3.6% |
| Anger | 0.84 | 0.85 | 0.85 | 0.81 | +4.9% |
| Sadness | 0.88 | 0.87 | 0.87 | 0.83 | +4.8% |
| Disgust | 0.83 | 0.79 | 0.81 | 0.76 | +6.6% |
| Shame | 0.82 | 0.78 | 0.80 | 0.74 | +8.1% |
| Guilt | 0.86 | 0.85 | 0.85 | 0.81 | +4.9% |

The results indicate that the proposed LER model achieves consistently higher F1-scores across all emotion categories compared to RoBERTa alone. The largest gains were observed in minority classes such as shame and disgust, which improved by 8.1% and 6.6%, respectively. These findings corroborate the effectiveness of the LSTM layer in modeling temporal dependencies and improving recognition of subtle emotional cues often underrepresented in the dataset. Table 11 presents the ablation on imbalance handling techniques (Macro-F1 on Validation Set).

**Table 11** Ablation on Imbalance Handling Techniques (Macro-F1 on Validation Set)

| **Technique** | **Macro-F1 (%)** | **Minority F1 Gain (%)** | **Training Time Increase (%)** | **Notes** |
| --- | --- | --- | --- | --- |
| Baseline (No Balancing) | 84.2 | - | 0 | Biased toward majorities |
| Class-Weighting | **86.9** | **+6.5** | <1 | Chosen; seamless integration |
| SMOTE (TF-IDF) | 85.7 | +4.1 | +25 | Noise in embeddings |
| Focal Loss | 86.5 | +5.9 | +8 | Comparable but tuning-heavy |

Furthermore, the high performance on dominant classes such as joy and sadness confirms that the hybrid LER framework maintains strong generalization while mitigating class imbalance through synonym-based augmentation. The relatively smaller gap between precision and recall values across classes suggests balanced performance, indicating the model’s robustness in distinguishing between semantically overlapping emotions.

1. Conclusion and Future Directions

This study introduces a new hybrid DL model based on LSTM and RoBERTa architectures to enhance the emotion detection accuracy within textual data. The study addresses such dominating challenges of this problem type, including context preservation and the complexity of sentence structures, while demonstrating dramatic improvements in terms of accuracy and robustness. With the sequential learning capability of LSTM and contextual embeddings generated by RoBERTa, the proposed model captures subtle emotional cues effectively, outperforming conventional methods on benchmark datasets. This research thus opens a promising pathway toward advancing NLP-based emotion recognition systems with applications across mental health diagnostics, sentiment analysis, and human-computer interaction. Future work will involve extending this model to perhaps an ensemble approach or integrating other context-aware mechanisms with greater adaptability and accuracy for different real-world settings.

For future enhancements, the model can be expanded with attention mechanisms and transformer-based encoder-decoder models to further improve emotion detection accuracy. Additionally, self-supervised learning and domain adaptation methods can be investigated for generalizing the model across different languages and domains with small amounts of labeled data. In terms of potential applications, the LER model promises much in real-world situations like sentiment analysis in social media surveillance, chat-based mental health evaluation, customer feedback assessment, and empathetic human-computer interaction systems. These uses assure the practical usefulness and scalability of the proposed solution in research and practice.

**Ethical Considerations**

This study makes use of the publicly available ISEAR dataset, which has been widely adopted in affective computing and emotion recognition research. All data used is fully anonymized and does not contain personally identifiable information (PII), ensuring compliance with ethical research standards. No new human subjects were recruited, and therefore, institutional review board (IRB) approval was not required. The analysis was conducted solely for academic and scientific purposes, with careful consideration to avoid misuse or misrepresentation of the emotional data. The findings are intended to advance research in natural language processing and should not be applied in high-stakes or sensitive domains without further ethical validation.

**Ethical Approval and Support**

Although this study involves analysis of emotional expressions derived from human subjects, it exclusively utilized the publicly available ISEAR dataset, which is fully anonymized and distributed for research purposes under open-access terms. No new data were collected directly from human participants, and no personally identifiable information (PII) is included. Consequently, this research did not require separate Institutional Review Board (IRB) or ethical committee approval.
However, to ensure ethical compliance, all data handling and analyses were conducted in accordance with the ethical principles outlined in the Declaration of Helsinki (2013) and institutional data governance standards. The dataset’s usage adheres strictly to its licensing terms and was approved for non-commercial academic research.

This ethical framework ensures that the study maintains integrity, transparency, and respect for data privacy while advancing emotion recognition research.

**Funding Declaration** This research work was funded by Institutional Fund Projects under grant no. (GPIP: 1046-611-2024). The authors gratefully acknowledge technical and financial support provided by the Ministry of Education and King Abdulaziz University, DSR, Jeddah, Saudi Arabia.

**Data Availability Statement:** The use in this study has been taken from the Kaggle repository online available at: https://www.kaggle.com/datasets/faisalsanto007/isear-dataset.

References

1. Abdel-Salam, S., & Rafea, A. (2022). Performance study on extractive text summarization using BERT models. Information, 13(2), 67.
2. Akingbesote, D., Zhan, Y., Maskeliūnas, R., & Damaševičius, R. (2023). Improving Accuracy of Face Recognition in the Era of Mask-Wearing: An Evaluation of a Pareto-Optimized FaceNet Model with Data Preprocessing Techniques. Algorithms, 16(6), 292.
3. Al-Qatf M, Lasheng Y, Al-Habib M, Al-Sabahi K (2018) Deep learning approach combining sparse autoencoder with SVM for network intrusion detection. IEEE Access 6:52843–52856
4. Al Maruf, A., Khanam, F., Haque, M. M., Jiyad, Z. M., Mridha, M. F., & Aung, Z. (2024). Challenges and opportunities of text-based emotion detection: a survey. IEEE access, 12, 18416-18450.
5. Alnuaim, A. A., Zakariah, M., Shukla, P. K., Alhadlaq, A., Hatamleh, W. A., Tarazi, H., ... & Ratna, R. (2022). Human‐computer interaction for recognizing speech emotions using multilayer perceptron classifier. Journal of Healthcare Engineering, 2022(1), 6005446.
6. Alroobaea R (2020) An Empirical combination of Machine Learning models to Enhance author profiling performance. International Journal of Advanced Trends in Computer Science and Engineering 9:2130–2137. https://doi.org/10.30534/ijatcse/2020/187922020
7. Alslaity, A., & Orji, R. (2024). Machine learning techniques for emotion detection and sentiment analysis: current state, challenges, and future directions. Behaviour & Information Technology, 43(1), 139-164.
8. Amin J, Sharif M, Raza M, et al (2019) Brain tumor detection using statistical and machine learning method. Computer Methods and Programs in Biomedicine 177:69–79. https://doi.org/10.1016/j.cmpb.2019.05.015
9. Asghar, M. Z., Subhan, F., Ahmad, H., Khan, W. Z., Hakak, S., Gadekallu, T. R., & Alazab, M. (2021). Senti‐eSystem: a sentiment‐based eSystem‐using hybridized fuzzy and deep neural network for measuring customer satisfaction. Software: Practice and Experience, 51(3), 571-594.
10. Ashraf MA, Nawab RMA, Nie F (2020) Author profiling on bi-lingual tweets. Journal of Intelligent and Fuzzy Systems 39:2379–2389. https://doi.org/10.3233/JIFS-179898
11. Asiri AA, Badshah A, Muhammad F, et al (2022) Human Emotions Classification Using EEG via Audiovisual Stimuli and AI. Computers, Materials and Continua 73:5075–5089. https://doi.org/10.32604/cmc.2022.031156
12. Botunac I, Brkić Bakarić M, Matetić M (2024) Comparing Fine-Tuning and Prompt Engineering for Multi-Class Classification in Hospitality Review Analysis. Applied Sciences 14:6254. https://doi.org/10.3390/app14146254
13. Cassab, S., & Kurdy, M. B. (2020). Ontology-based emotion detection in Arabic social media. Int. J. Eng. Res. Technol., 9(8), 1991-2013.
14. Chatterjee, A., Gupta, U., Chinnakotla, M. K., Srikanth, R., Galley, M., & Agrawal, P. (2019). Understanding emotions in text using deep learning and big data. Computers in Human Behavior, 93, 309-317.
15. Chen, T., Ju, S., Yuan, X., Elhoseny, M., Ren, F., Fan, M., & Chen, Z. (2018). Emotion recognition using empirical mode decomposition and approximation entropy. Computers & Electrical Engineering, 72, 383-392.
16. Chung, W., & Zeng, D. (2020). Dissecting emotion and user influence in social media communities: An interaction modeling approach. Information & Management, 57(1), 103108.
17. Chutia, T., & Baruah, N. (2024). Text-based Emotion Detection: A Review. International Journal of Digital Technologies, 3(I).
18. Dennison, J. (2024). Emotions: functions and significance for attitudes, behaviour, and communication. Migration studies, 12(1), 1-20.
19. Ekman, P. (1992). An argument for basic emotions. Cognition & emotion, 6(3-4), 169-200.
20. Ekman P (1999) Basic Emotions, vol. 476 of Handbook of Cognition and Emotion. T. Dalgleish and M. Power, John Wiley & Sons Ltd Sussex UK
21. Ezzameli K, Mahersia H (2023) Emotion recognition from unimodal to multimodal analysis: A review. Information Fusion 99:101847
22. Fontaine, J. R., Scherer, K. R., Roesch, E. B., & Ellsworth, P. C. (2007). The world of emotions is not two-dimensional. Psychological science, 18(12), 1050-1057.
23. Grissa D, Rasmussen DN, Krag A, et al (2020) Alcoholic liver disease: A registry view on comorbidities and disease prediction. PLoS Computational Biology 16:. https://doi.org/10.1371/journal.pcbi.1008244
24. Guo P, Liu T, Zhang Q, et al (2017) Developing a dengue forecast model using machine learning: A case study in China. PLoS Neglected Tropical Diseases 11:1–22. https://doi.org/10.1371/journal.pntd.0005973
25. Hammad M, Al-Awadi M (2016) Sentiment analysis for arabic reviews in social networks using machine learning. In: Information Technology: New Generations: 13th International Conference on Information Technology. Springer, pp 131–139
26. Hasan, M., Rundensteiner, E., & Agu, E. (2019). Automatic emotion detection in text streams by analyzing twitter data. International Journal of Data Science and Analytics, 7(1), 35-51.
27. Hossain, M. S., & Muhammad, G. (2019). Emotion recognition using deep learning approach from audio–visual emotional big data. Information Fusion, 49, 69-78.
28. Huang S, Nianguang CAI, Penzuti Pacheco P, et al (2018) Applications of support vector machine (SVM) learning in cancer genomics. Cancer Genomics and Proteomics 15:41–51. https://doi.org/10.21873/cgp.20063
29. Ikram ST, Cherukuri AK (2016) Improving accuracy of intrusion detection model using PCA and optimized SVM. Journal of Computing and Information Technology 24:133–148. https://doi.org/10.20532/cit.2016.1002701
30. Iqbal A, Aftab S, Ali U, et al (2019) Performance analysis of machine learning techniques on software defect prediction using NASA datasets. International Journal of Advanced Computer Science and Applications 10:300–308. https://doi.org/10.14569/ijacsa.2019.0100538
31. Jiang H, He Z, Ye G, Zhang H (2020) Network Intrusion Detection Based on PSO-Xgboost Model. IEEE Access 8:58392–58401. https://doi.org/10.1109/ACCESS.2020.2982418
32. Kas, M., El-merabet, Y., Ruichek, Y., & Messoussi, R. (2022). Generative adversarial networks for 2D-based CNN pose-invariant face recognition. International Journal of Multimedia Information Retrieval, 11(4), 639-651.
33. Khan B, Naseem R, Muhammad F, et al (2020) An empirical evaluation of machine learning techniques for chronic kidney disease prophecy. IEEE Access 8:55012–55022. https://doi.org/10.1109/ACCESS.2020.2981689
34. Khan S, Ullah R, Khan A, et al (2016) Analysis of dengue infection based on Raman spectroscopy and support vector machine (SVM). Biomedical Optics Express 7:2249. https://doi.org/10.1364/boe.7.002249
35. Kovács G, Balogh V, Mehta P, et al (2019) Author profiling using semantic and syntactic features notebook for PAN at CLEF 2019. CEUR Workshop Proceedings 2380:9–12
36. Kumar, S. H., Vijay, T. A., Thirukumaran, P., & Balaji, V. B. (2023, June). Intrusion Detection System based on Pattern Recognition using CNN. In 2023 International Conference on Sustainable Computing and Smart Systems (ICSCSS) (pp. 567-574). IEEE.
37. Lee, P., Teng, Y., & Hsiao, T. C. (2012, July). XCSF for prediction on emotion induced by image based on dimensional theory of emotion. In Proceedings of the 14th annual conference companion on Genetic and evolutionary computation (pp. 375-382).
38. Liu J, Kantarci B, Adams C (2020) Machine learning-driven intrusion detection for Contiki-NG-based IoT networks exposed to NSL-KDD dataset. WiseML 2020 - Proceedings of the 2nd ACM Workshop on Wireless Security and Machine Learning 25–30. https://doi.org/10.1145/3395352.3402621
39. Machado S, Ribeiro AC (2019) Machine learning algorithms and techniques for sentiment analysis in scientific paper reviews: A systematic literature review
40. Mehr, S. Y., & Ramamurthy, B. (2019, December). An SVM based DDoS attack detection method for Ryu SDN controller. In Proceedings of the 15th international conference on emerging networking experiments and technologies (pp. 72-73).
41. Mehrabian, A. (1996). Pleasure-arousal-dominance: A general framework for describing and measuring individual differences in temperament. Current Psychology, 14, 261-292.
42. Menzies T, Dekhtyar A, Distefano J, Greenwald J (2007) Problems with precision: A response to “Comments on ‘data mining static code attributes to learn defect predictors.’” IEEE Transactions on Software Engineering 33:637–640. https://doi.org/10.1109/TSE.2007.70721
43. Muslim, T. O., Ahmed, A. N., Malek, M. A., Abdulmohsin Afan, H., Khaleel Ibrahim, R., El-Shafie, A., ... & El-Shafie, A. (2020). Investigating the influence of meteorological parameters on the accuracy of sea-level prediction models in Sabah, Malaysia. Sustainability, 12(3), 1193.
44. Narendra, B., Sai, K. U., Rajesh, G., Hemanth, K., Teja, M. C., & Kumar, K. D. (2016). Sentiment analysis on movie reviews: a comparative study of machine learning algorithms and open source technologies. International Journal of Intelligent Systems and Applications (IJISA), 8(8), 66.
45. Naseem R, Khan B, Ahmad A, et al (2020) Investigating Tree Family Machine Learning Techniques for a Predictive System to Unveil Software Defects. Complexity 2020:1–21. https://doi.org/10.1155/2020/6688075
46. Olaniyan, D., Ogundokun, R. O., Bernard, O. P., Olaniyan, J., Maskeliūnas, R., & Akande, H. B. (2023). Utilizing an attention-based LSTM model for detecting sarcasm and irony in social media. Computers, 12(11), 231.
47. Pham BT (2018) A Novel Classifier Based on Composite Hyper-cubes on Iterated Random Projections for Assessment of Landslide Susceptibility. Journal of the Geological Society of India 91:355–362. https://doi.org/10.1007/s12594-018-0862-5
48. Plutchik, R. (1980). A general psychoevolutionary theory of emotion. In Theories of emotion (pp. 3-33). Academic press.
49. PS, S., & Mahalakshmi, G. (2017). Emotion models: a review. International Journal of Control Theory and Applications, 10(8), 651-657.
50. Pumpuang P, Srivihok A, Praneetpolgrang P (2008) Comparisons of classifier algorithms: Bayesian network, C4.5, Decision Forest and NBTree for Course Registration Planning Model of undergraduate students. Conference Proceedings - IEEE International Conference on Systems, Man and Cybernetics 3647–3651. https://doi.org/10.1109/ICSMC.2008.4811865
51. Rau HH, Hsu CY, Lin YA, et al (2016) Development of a web-based liver cancer prediction model for type II diabetes patients by using an artificial neural network. Computer Methods and Programs in Biomedicine 125:58–65. https://doi.org/10.1016/j.cmpb.2015.11.009
52. Rodríguez, A. O. R., Riaño, M. A., García, P. A. G., Marín, C. E. M., Crespo, R. G., & Wu, X. (2020). Emotional characterization of children through a learning environment using learning analytics and AR-Sandbox. Journal of Ambient Intelligence and Humanized Computing, 11, 5353-5367.
53. Russell, J. A. (1980). A circumplex model of affect. Journal of personality and social psychology, 39(6), 1161.
54. Sailunaz, K., Dhaliwal, M., Rokne, J., & Alhajj, R. (2018). Emotion detection from text and speech: a survey. Social Network Analysis and Mining, 8(1), 28.
55. Sboev A, Litvinova T, Gudovskikh D, et al (2016) Machine Learning Models of Text Categorization by Author Gender Using Topic-independent Features. Procedia Computer Science 101:135–142. https://doi.org/10.1016/j.procs.2016.11.017
56. Shah, P., & Swaminarayan, P. (2022). Machine learning-based sentiment analysis of Gujarati reviews. International Journal of Data Analysis Techniques and Strategies, 14(2), 105-121.
57. Singh, M., Jakhar, A. K., & Pandey, S. (2021). Sentiment analysis on the impact of coronavirus in social life using the BERT model. Social Network Analysis and Mining, 11(1), 33.
58. Singh, S. K., Verma, P., & Kumar, P. (2020). Sentiment analysis using machine learning techniques on twitter: a critical review. Adv. Math.: Scientific Journal, 9(9), 7085-7092.
59. Tran, V. K., & Nguyen, L. M. (2017, August). Semantic refinement gru-based neural language generation for spoken dialogue systems. In International Conference of the Pacific Association for Computational Linguistics (pp. 63-75). Singapore: Springer Singapore.
60. Vakili, M., Ghamsari, M., & Rezaei, M. (2020). Performance analysis and comparison of machine and deep learning algorithms for IoT data classification. arXiv preprint arXiv:2001.09636.
61. Vaseeharan, T., & Aponso, A. (2020, February). Review on sentiment analysis of twitter posts about news headlines using machine learning approaches and naïve bayes classifier. In Proceedings of the 2020 12th International Conference on Computer and Automation Engineering (pp. 33-37).
62. Velagalet, S. B., Choukaier, D., Nuthakki, R., Lamba, V., Sharma, V., & Rahul, S. (2024). Empathetic algorithms: The role of ai in understanding and enhancing human emotional intelligence. Journal of Electrical Systems Inc, 20, 2051-2060.
63. Waelen, R. (2024). Philosophical lessons for emotion recognition technology. Minds and Machines, 34(1), 3.
64. Wang, Q., Xu, C., Zhou, Y., Ruan, T., Gao, D., & He, P. (2018, December). An attention-based Bi-GRU-CapsNet model for hypernymy detection between compound entities. In 2018 IEEE International Conference on Bioinformatics and Biomedicine (BIBM) (pp. 1031-1035). IEEE.
65. Wang, W., Chen, L., Thirunarayan, K., & Sheth, A. P. (2012, September). Harnessing twitter" big data" for automatic emotion identification. In 2012 International Conference on Privacy, Security, Risk and Trust and 2012 International Confernece on Social Computing (pp. 587-592). IEEE.
66. Watson D, Clark LA, Tellegen A (1988) Development and validation of brief measures of positive and negative affect: the PANAS scales. Journal of personality and social psychology 54:1063
67. Xu, D., Tian, Z., Lai, R., Kong, X., Tan, Z., & Shi, W. (2020). Deep learning based emotion analysis of microblog texts. Information Fusion, 64, 1-11.
68. Zhu, Z., Dai, W., Hu, Y., & Li, J. (2020). Speech emotion recognition model based on Bi-GRU and Focal Loss. Pattern Recognition Letters, 140, 358-365.
